# Implementation of Green Lean Six Sigma in Dutch Healthcare: A Qualitative Study of Healthcare Professionals’ Experiences

**DOI:** 10.3390/nursrep14040210

**Published:** 2024-10-09

**Authors:** Marieke Sijm-Eeken, Annick Greif, Linda Peute, Monique Jaspers

**Affiliations:** 1 Department of Medical Informatics, Amsterdam UMC Location University of Amsterdam, Meibergdreef 9, 1105 AZ Amsterdam, The Netherlands; 2 Amsterdam UMC Center for Sustainable Healthcare, Postbus 22660, 1100 DD Amsterdam, The Netherlands; 3 Amsterdam Public Health Research Institute, De Boelelaan 1117, 1081 HV Amsterdam, The Netherlands; 4 Center for Human Factors Engineering of Health Information Technology, Meibergdreef 9, 1105 AZ Amsterdam, The Netherlands

**Keywords:** GLSS, training evaluation, barriers, healthcare, sustainability, nursing

## Abstract

**Introduction:** The healthcare sector significantly contributes to environmental degradation, highlighting the need for sustainable practices. Green Lean Six Sigma (GLSS) offers a relevant and impactful approach to reduce healthcare’s environmental footprint while improving efficiency. By incorporating environmental considerations into Lean Six Sigma, GLSS has the potential to mitigate healthcare’s environmental impact and promote environmental sustainability. This study aims to gain insight into healthcare professionals’ experiences with GLSS at their workplace. **Materials and Methods**: This qualitative exploratory study employed semi-structured surveys based on theory of training evaluation from Kirkpatrick with Dutch healthcare professionals in the first six to eight months after completing GLSS training. **Results:** Even though 76% (*N* = 16) of trained healthcare professionals applied GLSS at their workplace and 43% of them (*N* = 9) completed a project within the first six to eight months after training, they all experienced one or more barriers. The most frequently reported barriers were lack of time, difficulties with project selection and a lack of management support. GLSS project results included reduction of products, energy, costs and travel, green choices in procurement and sustainable food choices. GLSS also helped to create awareness on the environmental impact of healthcare and to optimize processes by reducing costs, waiting time, workload and defects. **Discussion**: This is the first study to report experiences from applying GLSS in healthcare. Furthermore, it is the first study presenting GLSS training evaluation results in terms of participant behaviour and organizational outcomes. **Conclusions**: Results of this study can be used to enhance GLSS deployment programs and to optimize organizational settings for successful GLSS implementation in healthcare.

## 1. Introduction

The healthcare sector exerts a substantial influence on the environment and stands out as one of the industries with the highest levels of pollution [1]. Given the present sense of urgency surrounding climate issues and the imperative to minimize the environmental impact of healthcare, scholars and practitioners from various fields, including healthcare, are actively exploring novel approaches and solutions. The Green Lean Six Sigma (GLSS) methodology has emerged as a potentially effective approach for addressing healthcare’s environmental impact and mitigating climate change [2,3].

In GLSS, environmental sustainability principles and tools are merged with the Lean Six Sigma (LSS) methodology [4]. The LSS methodology has gained significant adoption and demonstrated effectiveness across various industries, including healthcare [5,6]. The application of LSS in the healthcare sector has previously been associated with various conventional advantages including the reduction of process lead times and costs, enhancement of patient satisfaction, and improvement of patient health outcomes [5]. The original examination of the combination of LSS and environmental sustainability was mostly conducted in a theoretical and conceptual manner [7,8]. However, as time progressed, GLSS was acknowledged and put into use in practice. For example, the incorporation of sustainability measures into the process of value stream mapping, commonly known as “environmental value stream mapping” or “E-VSM”, was discussed in previous studies [9,10]. As GLSS projects have been implemented and carried out, various case studies offered insight into success factors, barriers, and other facets of the approach, which contributed to the development of implementation models [11]. An example of an implementation model in healthcare is the model from Trakulsunti et al. [2], who provided a specific approach for implementing GLSS to reduce medication errors.

Despite the potential promise of GLSS in mitigating the environmental impact of healthcare, the available research about its practical implications within the healthcare industry remains limited. In a study conducted by Sijm-Eeken et al. [12], a novel training program for healthcare professionals focusing on GLSS was designed. Findings from the program’s preliminary evaluation focused on participants’ initial response to the training and their level of knowledge acquisition and confidence to apply theory into practice, indicated favorable outcomes. At the time the aforementioned evaluation was conducted, it was too early to determine how participants of the training experienced the deployment of GLSS projects in their workplace healthcare setting and what results they were able to realize.

Without a comprehensive understanding of the effects of GLSS implementation in the healthcare sector, the potential benefits of GLSS in healthcare remain unknown. Furthermore, in the absence of such understanding, it is uncertain how the theoretical underpinnings of GLSS could be augmented to enhance its efficacy. Questions such as: “Which specific environmental impacts can be reduced by using GLSS?”, ‘What are the primary barriers encountered by GLSS practitioners when applying the methodology?’ and ‘To what extend are concepts and tools from GLSS useful in GLSS projects?’ remain to be answered.

The main aim of this study is to explore the firsthand experiences and viewpoints of healthcare professionals about the real-world application of GLSS through project-based implementation. The study’s objectives are to identify barriers to GLSS implementation, assess the perceived value and effectiveness of GLSS and to contribute to GLSS knowledge and practice. To accomplish these objectives, the present research examines the barriers for successful execution of GLSS projects within the healthcare industry by means of a survey with healthcare professionals who completed GLSS training. Additionally, the survey was used to investigate the perceived value and efficacy of GLSS concepts and tools in healthcare GLSS projects from the perspective of practitioners.

The findings of this study have the potential to support researchers, trainers and practitioners in the field of GLSS in healthcare in their efforts to enhance the theoretical and practical foundations related to GLSS, hence enhancing the implementation of GLSS projects. To our knowledge, this is the first study in which the application of GLSS in healthcare practice is evaluated.

This article first describes the study’s context, followed by an outline of the methodology including the development of the survey used to collect responses from training participants after the training. Additionally, it provides detailed information on the specific projects undertaken by the participants and how these projects contributed to their overall application of GLSS principles

## 2. Study Context

The research outlined in this paper represents a component of a longitudinal study. The study involves the development and implementation of a GLSS training program for healthcare professionals in the Netherlands. The training program at the Greenbelt level was specifically designed for healthcare professionals of various roles and is most commonly referred to as the “LSS sustainable healthcare training”. The training program incorporates standardized content for Green Belt training from the recognized international LSS body, the American Society for Quality (ASQ), supplemented with tools and examples specifically focused on the environmental impacts of healthcare. The focus of the training lies in the utilization of the GLSS methodology to address and minimize the environmental impacts of healthcare activities across a broad set of domains. This encompasses the pollution of water, air, and soil, as well as the generation of waste and utilization of resources [13]. Moreover, within the scope of the GLSS training program, the concept of “waste” was broadened to encompass avoidable environmental consequences, in addition to the conventional understanding of waste in LSS. Consequently, the DOTWIMP tool underwent an expansion to address the evaluation of product utilization, including disposable products, packaging, and food next to its common sources of waste. The preceding publication by Sijm-Eeken et al. [12] provides a narrative of the training development and initial evaluation outcomes. An overview of the training organization and its content can be found in Appendix A.

## 3. Materials and Methods

### 3.1. Methodological Overview

Surveys provide a structured means of gathering qualitative data from participants, supporting the analysis, understanding and evaluation of training and practical experiences [13]. A qualitative research approach was followed to meet this study’s aim to identify and understand experiences with implementing GLSS in Dutch healthcare organizations.

All 39 individuals who commenced the GLSS training program, provided their active written informed consent to participate in this study. Baseline data were collected for these individuals in the prior study by Sijm-Eeken et al. [12]. Subsequently, an initial evaluation of the GLSS training was conducted upon completion, involving the participation of 29 healthcare professionals who successfully completed the training.

The multiple GLSS initiatives and projects these participants had started after taking the training and the context they were deployed in (including the participant and the healthcare setting) are the anticipated units of analysis in this qualitative exploratory study. All 29 training graduates were requested to participate in the present study six to eight months following the end of the training. In the longitudinal study of which this research is a part, the initial data collection phase prior to the training is denoted as “t_0_”, the immediate post-training feedback collection phase is denoted as “t_1_”, and the data collection phase occurring six to eight months after training completion is denoted as “t_2_”. The assessment in this study is predicated upon the data collected at time point t_0_ and t_1_ to facilitate a comprehensive analysis of the outcomes at time point t_2_. To ensure clarity and comprehensiveness, Appendix B describes the data collected on t_0_ and t_1_ data.

The subsequent section provides a description of the methodology employed in constructing the survey utilized to gather the responses from participants of their implementation of GLSS in their work environments at t_2_. The evaluations of the training on t_1_ and t_2_ are grounded in the four-level training evaluation model developed by Kirkpatrick and Kirkpatrick [14] because this model is widely recognized and used for training evaluation. The first evaluation of the GLSS training on t_1_ was conducted using Kirkpatrick and Kirkpatrick’s lowest two levels, Level 1 “Reaction” and Level 2 “Learning”, respectively. The evaluation of the training on t_2_ in the current study is based upon the highest two levels (Level 3 “Behaviour” and Level 4 “Results”). The Behaviour level assesses the degree to which individuals implement the knowledge and skills acquired by the training within their respective work environments. Level 4, Results, evaluates outcomes of applying knowledge and skills in order to ascertain the efficacy of training programs [14].

**Figure 1:**
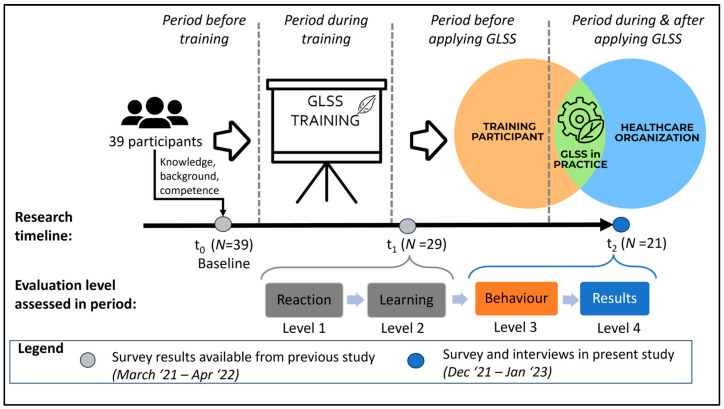
Overall study design. *Activities till t_1_ (marked in grey color) were subject of previous study [12]. Evaluation levels are based on Kirkpatrick & Kirkpatrick [14]*.

The overall study design is summarized in Figure 1, where the left side relates to previously completed research and the right side (starting at time t_1_ and ending at time t_2_) represents the research presented in this paper.

### 3.2. Construction of Survey

As suggested by Maltby et al. [13], a survey was utilized to gather information on the experiences from participants six to eight months after the training. The theoretical framework proposed by Kirkpatrick and Kirkpatrick [14] for evaluating the levels of Behaviour and Results following training completion appeared relevant to the aims of this study. However, the existing literature lacked a standardized and validated questionnaire for evaluating adult training programs at these levels. Therefore, the research team devised instruments by modifying a pre-existing questionnaire that is grounded in Kirkpatrick’s model for evaluating adult training. The aforementioned questionnaire was formulated and tested by Alsalamah and Callinan [15] and is the sole questionnaire derived from the implementation of the search strategy outlined in Appendix C.

The research team, which included an LSS expert and trainer, evaluated behavior-level questions for their relevance to GLSS evaluation at training participants’ workplaces. Kirkpatrick’s model and criteria for behavior-level training evaluation [16] were used to complete the questionnaire. The resulting questions make up the first and last section of the questionnaire (Part A and Part C). Which questions are appropriate for assessing outcomes on the Results level is distinct for each training program and depends upon the training objectives [14]. In the case of this study these objectives relate to the outcomes of applying GLSS at the workplace of training participants. The World Health Organization (WHO) guidance for climate-resilient and environmentally sustainable healthcare facilities [17] was used to structure the questions on outcomes in Part B of the questionnaire. Not only do these guidelines focus on the environmental impact of healthcare facilities—it also provides a structured framework in which improvements can be classified. Several conventional LSS project outcomes (reductions of time, costs, errors/mistakes and workload) were added to facilitate the identification of synergies in environmental and other project outcomes. Additionally, a question was constructed regarding the GLSS tools employed in these projects so that the research team could gain insights into the tools that were already used by the participants and the tools that they expect to be using in the future. Appendix D contains the original questions developed by Alsalamah and Callinan [15], revisions made, and additional questions resulting from the review process that produced the final questionnaire.

The resulting questionnaire was pretested by one training participant. This pretest assessed question clarity and questionnaire completion time. One question was removed as a result of this pretest to shorten the survey and prevent overlap.

### 3.3. Data Collection and Case Description

The survey data was gathered between July to October 2022 by requesting all 29 participants who successfully completed the training to complete the survey. A subset of 21 persons completed the survey six to eight months after completing the course. These 21 participants were selected and included in the current study. Table 1 presents a summary of the demographic characteristics and self-reported previous experience of the individuals involved in the study.

Participants were employed in eight distinct healthcare organizations, in a variety of roles. Ten individuals were directly involved in patient care, with two of them serving as nurses and five as medical physicians. Other participants were project manager, process manager, and process operator in a range of departments, including procurement, logistics, waste management, catering, and retail, as well as laboratory and quality management.

Besides the survey data, information from the trainer resulting from project reviews was used to assess the level of completeness of GLSS projects.

### 3.4. Data Analysis

The survey results and participant data gathered at time point t_0_ and t_1_ were consolidated in a spreadsheet and subjected to analysis using Minitab 17 Statistical Software. Because of the small sample size (total population size consisting of 29 participants), the application of qualitative analysis was limited to testing on significant differences between groups of respondents, depending on the level of GLSS application. To this end, respondents were distributed into three groups based on their achievements with applying GLSS in practice: (i) participants who did not apply GLSS (ii) participants who applied GLSS without completing a project (iii) participants who finished a GLSS project. To examine relationships between demographics, background characteristics, and survey responses on one hand and the level by which participants were able to apply GLSS (defined by the afore mentioned three groups) on the other hand, we employed ANOVA tests on ordinal data and Chi-square tests on nominal variables. Normality and homogeneity of variances for variables were confirmed prior to ANOVA testing by the Ryan-Joiner and Levene’s tests. These quantitative ANOVA and Chi-square tests were used to complement the qualitative data.

The survey responses from the open questions were retrieved by researcher AG. Subsequently, MSE and AG collaborated to cluster the recovered responses. Particular emphasis was placed on the identification of barriers encountered by the participants in their efforts to implement GLSS, because it was one of the objectives of the research to identify the barriers experienced by healthcare professionals.

## 4. Results

### 4.1. Project Initiation and Completion

The 21 participants who responded to the survey were categorized into three distinct groups according to their level of application of the GLSS methodology at their respective workplaces: those who had not applied it at all, those who had applied it but had not completed a project, and those who had successfully completed a GLSS project. Table 2 provides an overview of the demographics, background information and participant information collected before the training.

Table 3 presents the respondents survey results directly after completing the training and Table 4 summarizes the survey results collected six to eight months after the training for each of these three groups.

There is no evidence of significant differences observed in the demographics, background characteristics, and survey responses provided by participants among the three groups. Nevertheless, the use of GLSS tools by persons within the project-completing group is notably greater compared to the tool usage by members in the other groups.

### 4.2. Evaluation of GLSS Tools and Concepts

**Figure 2:**
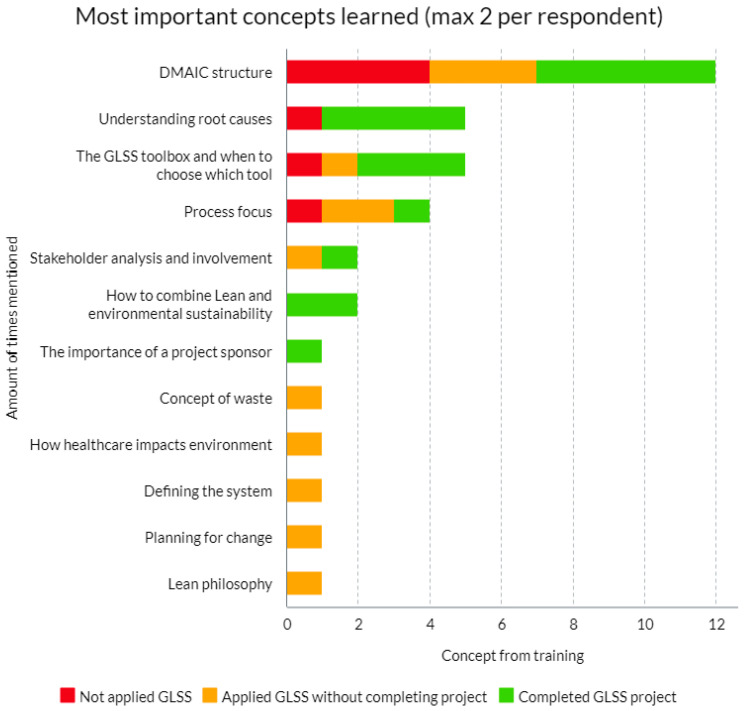
Answers to question on two major concepts learned.

The concepts mentioned by respondents when asked to identify the two major concepts learned during the GLSS training and their frequency are presented in Figure 2.

The respondents from all three groups most frequently mentioned the DMAIC framework, which encompasses the Define, Measure, Analyze, Improve, and Control phases of the Lean Six Sigma approach. Responses grouped within this category include explicit references to the DMAIC framework and answers pertaining to the “structured Lean Six Sigma approach”, and comparable responses. Understanding root causes, the GLSS toolbox as a whole and process focus were also mentioned by a minimum of four respondents.

Table 5 presents a comprehensive overview of the LSS tools that were introduced throughout the training course. The colors serve as indicators of the participant’s experienced and perceived relevance of the tool, based on their actual and intended application of the tool The data indicates that the Project Charter and Improvement Plan were the most frequently applied tools, being referenced 11 and 8 times respectively, followed by the Stakeholder analysis and the SIPOC. While most tools are either already used or strongly expected to be used in the future by the majority of the participants, this does not apply to the Critical to Quality Tree, Process Mapping, DOTWIMP, 5S, Value Add Analysis, and Statistical Analysis tools. The tool that is most frequently assessed by participants as a tool they do not expect to use in the future is the Value Add Analysis. One participant (No. 19) did not use any tools and expressed uncertainty over their potential use in future endeavors.

One participant suggested adding another tool to the GLSS toolset: MECE (Mutually Exclusive Collectively Exhaustive).

In addition to the benefits of the GLSS approach, participants reported additional benefits from obtaining the training. These benefits include learning about best practices and examples from other organizations, making new connections with people who can be contacted for help and collaboration, identifying peers for support within their own organization, analyzing healthcare and non-healthcare scenarios, evaluating the environmental impacts of healthcare practices, and fostering awareness and effective communication.

### 4.3. Evaluation on Results Level

Achievements realized by applying learnings from the GLSS training as mentioned by participants are listed in Table 6.

This table shows that participants used GLSS to realize benefits for the environment, to create awareness among colleagues on the environmental impact and for realizing other benefits for their organization such as cost reduction, reduction of waiting time, reducing workload of employees, and reducing errors/mistakes.

**Figure 3:**
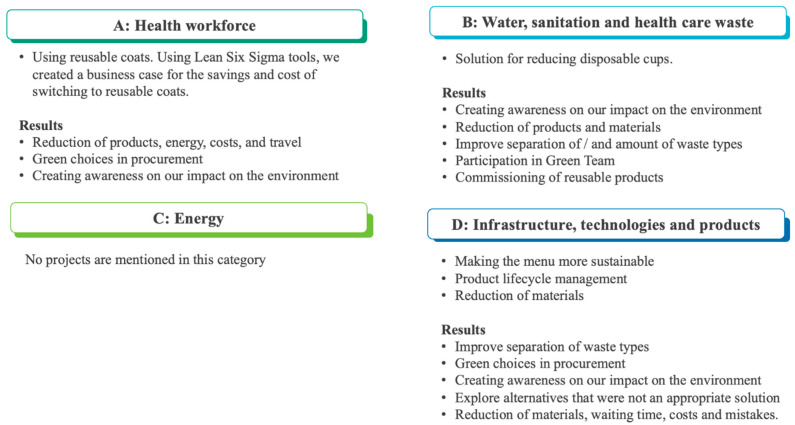
Participants projects and results categorized according to the environmental framework of Prats [17].

With regard to the environmental domain of the projects, Figure 3 describes the projects and results with the corresponding category according to the environmental framework of Prats [17].

### 4.4. Experienced Barriers and Success Factors

**Figure 4:**
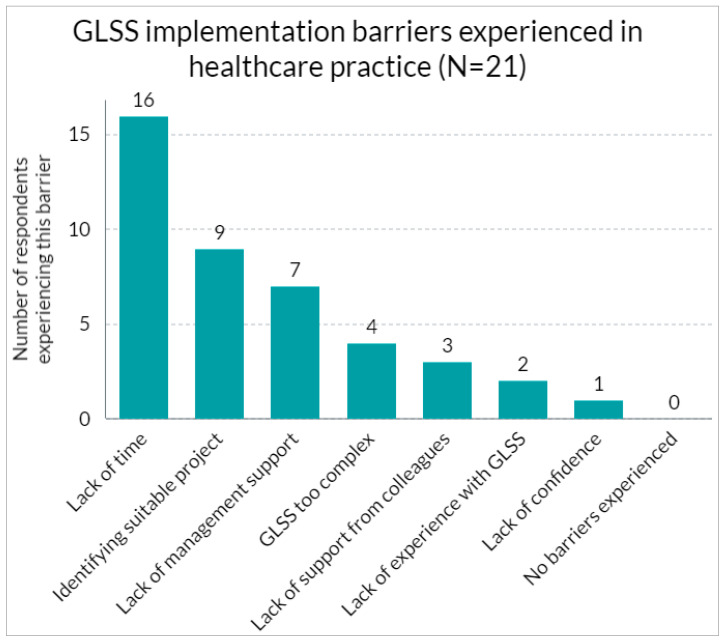
Barriers experienced when trying to apply GLSS in healthcare practice.

All participants reported experiencing challenges while attempting to implement or actually applying the GLSS method within their respective professional environments. Figure 4 illustrates the barriers encountered by the participants, together with the corresponding frequency of their mention.

The most common barrier healthcare professionals listed was “not having enough time” (76%, *N* = 16). This was followed by “choosing a good project to work on” (43%, *N* = 9), “not having enough support from management” (33%, *N* = 7), “the difficulty of GLSS” (19%, *N* = 4), “not having enough support from coworkers” (14%, *N* = 3), “not having enough experience with the GLSS method” (10%, *N* = 2), and “not having enough confidence to use GLSS” (5%, *N* = 1).

In addition to the GLSS tools and concepts, participants of the training reported that they had used additional knowledge and skills in their professional roles. The most frequently mentioned topic here is the concept of “reducing, reusing, and recycling” (*N* = 6). Furthermore, “finding peers that can help” was highlighted (*N* = 4), where one participant reported to have started a Green Team after connecting with peers. The knowledge on how healthcare impacts the environment (*N* = 2) was also provided as an answer to the question which other learnings from the training had been applied at the participants’ workplaces. Other answers included “using a Lean improvement board with colleagues to share barriers and successes and find improvements” (*N* = 1), “setting specific goals to address problems” (*N* = 1), “the importance of stakeholders and communication” (*N* = 1), “think before you act” (*N* = 1), and “starting a recycling program” (*N* = 1).

Sixteen respondents gave suggestions for improving the GLSS training. Firstly, it was recommended to conduct the training in a classroom setting rather than online (*N* = 3). Additionally, it was suggested to incorporate more practical tests alongside the existing quizzes that follow each theoretical section (*N* = 2). Furthermore, the inclusion of online group lessons was proposed to facilitate knowledge testing, interaction, and discussion among peers, thereby reducing the reliance on self-study (*N* = 3). To enhance the practical understanding of the subject matter, it was advised to provide more examples of practical Green Lean Six Sigma (GLSS) applications (*N* = 3). Moreover, it was suggested to streamline the training content by reducing the number of tools explained (*N* = 1). In terms of language proficiency, it was recommended to improve the quality of English speaking during online lessons (*N* = 1) and to provide subtitles for Dutch participants (*N* = 1). To ensure the retention of knowledge, the implementation of a refresher course after six to twelve months was proposed (*N* = 1). Lastly, it was suggested to develop a condensed version of the training specifically designed for project members, thereby relieving Green Belt participants from the burden of repeatedly explaining the core GLSS concepts (*N* = 1).

## 5. Discussion

This study aimed to gain insights into the perceived value of elements of the GLSS methodology, the realized benefits and barriers experienced by healthcare professionals while applying GLSS to mitigate environmental impact of healthcare after completing GLSS training.

The survey instrument was developed by adapting an existing training evaluation questionnaire, with additional questions tailored specifically to the GLSS content and training objectives. Of the 29 participants, 21 completed the survey, resulting in a response rate of 72.4%. This response rate falls slightly below the 75% threshold deemed acceptable for healthcare professional surveys, as suggested by Cook et al. [18]. Consequently, caution is warranted when generalizing these findings. However, some insights regarding the application of GLSS within healthcare practice can be extracted from the survey responses.

First, survey responses revealed that a majority of recently trained healthcare professionals were able to apply GLSS concepts and tools at their workplace in the first six to eight months after completing a GLSS training, even though they all experienced one or more barriers. This finding is in line with results presented by Yadav et al. [19] who performed a systematic review on the application of GLSS across different industries. They found that GLSS implementation leads to improvement in all sustainability areas.

Several frequently experienced barriers could also be extracted from the survey responses. The most often reported barriers include lack of time, identifying a suitable project to work on, and the lack of management support. These common Lean Six Sigma barriers were also identified by other researchers to be influencing the success of GLSS implementation [9,11,20,21,22,23]. The three factors: “GLSS being too complex”, “Lack of experience with GLSS” and “Lack of confidence” were barriers identified in our study that were not identified in these other studies on success factors for GLSS implementation. This could be explained by the fact that the participants in our study were new to GLSS and therefore not experienced in the methodology, while in other studies there was already more knowledge and expertise available in the organizations and individuals applying GLSS. Additionally, our study focused on the experiences of GLSS project implementation rather than deploying organization-wide programs based on GLSS focused on making GLSS part of the culture and strategy.

Letchumanan et al. [21] found “GLSS tools and techniques for effective data collection and measurement” to be a key success factor in seven studies, while this was not mentioned as a barrier by the participants of our study. This could be explained by the attention paid to measurement and the inclusion of measurable impact factors in the GLSS training materials in the GLSS training. Specifically, the HEIF-scheme [24] was offered in the training to support the data collection and measurement related to environmental impact factors.

Further, an interesting finding based on the survey outcomes is that, apart from process changes aimed at reducing environmental impact, participants indicated they applied learnings of GLSS to create awareness on the impact on the environment in their organizations. Awareness of the current environmental impact of healthcare organizations can help convince people that change is needed and therefore be a driver for change [25]. This effect of the training might help promote the use of green practices and encourages individuals to adopt more sustainable behaviors even outside of the training participants.

Other benefits for their healthcare organization mentioned by participants were cost reduction, reduction of process waiting time, reducing workload of employees and reducing errors/mistakes. This suggest that GLSS benefits can extend beyond environmental improvements. This is not an unexpected finding, because GLSS is based on the traditional LSS method and the afore mentioned benefits are common LSS improvement outcomes [26].

Our findings on the tools used by respondents also yield some interesting aspects. The fact that the Project Charter was used by most participants is in line with our expectation. The Project Charter is a prevalent tool in the initiation of LSS initiatives [25] and hence even those respondents who did not complete a project would benefit from using it. However, the unexpected aspect lies in the limited use of the Critical to Quality Tree tool, which was applied by only two participants. The GLSS training presented this tool as an important means for better understanding the “Voice of the customer”. Its limited use by participants of the training raises the question if sufficient attention has been given to potential negative impacts of solutions to reduce environmental impact on other aspects of quality of care such as patient health outcomes. Additionally, Process Mapping is considered an important step in GLSS but was applied by only one participant. A possible explanation for the limited application of Process Mapping could be that the GLSS projects were managed by persons who had extensive experience and knowledge of the processes. Combined with the lack of time to work on the improvement initiative which was a frequently report barrier for implementation of GLSS, this could have resulted in the limited application of Process Mapping.

According to the review performed by Jin et al. [27] which was published after the completion of our study, GLSS frameworks in the service sector are still in their early stage of development and should be tested on a wider scale to be able to review their effectiveness. Our research contributes to performing this testing in the specific setting of Dutch healthcare.

### Limitations

Even though the findings of this study are based on a limited sample size based on only the Dutch healthcare setting, the inclusion of participants with different roles and backgrounds working in diverse healthcare organizations helps to increase generalizability to the broader healthcare context. When extrapolating the findings to healthcare settings outside of the Netherlands, it is important to consider differences in environmental policies, practices and circumstances compared to the Netherlands including waste management habits and procurement standards (reusable versus disposable product preferences). An important factor influencing the results of this study is that the approach to GLSS used in the training was based on a specific selection of concepts and tools [12]. Since the core of the training was based on the international standard for Green Belt training from American Society for Quality [28], this mostly applies for the selection of “Green” tools and examples that were added to this standard to include the environmental focus. Furthermore, a specific design and delivery of the training itself was used. For example, research from Kumari et al. [29] found that online training is less effective than classroom training, therefore results of applying GLSS by participants of a classroom training could be more positive than the ones investigated in our research. Having said this, healthcare professionals are often busy, and lack of time was also found to be a key problem for GLSS implementation experienced by the participants, therefore a classroom training could result in less healthcare professionals being trained. Furthermore, this study can be impacted by non-response bias. Out of 40 trained healthcare professionals, 21 responded to the survey. Non-respondents might have been less successful with implementing GLSS at their workplace. However, if all non-respondents did not apply GLSS, still 40% of trained people applied one or more tools or concepts of GLSS.

We assessed the status six to eight months after receiving training. If assessed later, there could be more results. However, a study from Laux et al. [30] on the duration of typical LSS projects found an average actual duration of 4.2 months and that therefore, collecting data from participants six to eight months after the training should allow sufficient time for completing a project.

There are several areas that we suggest for future research which can contribute to further expanding the understanding on how GLSS can be applied for reducing the environmental impact of healthcare and how to optimize GLSS training. Apart from expanding the research to other countries than the Netherlands, it would be good to investigate the conditions on when GLSS is a suitable methodology to apply compared to other methods.

Findings of the present study indicated potential for GLSS projects for reducing environmental impact in settings where no “GLSS culture” or organization-wide deployment and adoption of GLSS were present. Because Letchumanan et al. [21] found that organization-wide deployments bring significant advantages and increase success, there could be even more potential of applying GLSS in such situations. Future studies on organization-wide deployment of GLSS in healthcare besides project-based deployment are therefore recommended.

Another area for future research relates to the selection of GLSS as a method for improvement compared to other methods. Even though findings of this study suggest that training healthcare professionals in GLSS can result in successful implementation for reducing the environmental impact of healthcare, we do not assume that GLSS is the best method to apply in all situations. This study showed that the method helped in understanding root causes and identifying “hot spots” for improving healthcare’s impact on the environment. However, when solutions are clear it would not be necessary to apply the full GLSS method and instead an implementation project would be sufficient and more efficient. Therefore, we expect that solutions implemented by GLSS projects can be adopted and further deployed in other healthcare settings without performing a full GLSS project. An important next step is to compare implementation of GLSS with other improvement methods in order to investigate for which settings GLSS is most suitable and where other methods are a better choice.

Lastly, to develop a full picture on how GLSS can be applied for reducing the environmental impact of healthcare, additional studies will be needed that follow-up participants of the training on the longer term, e.g., during several years after completing the training. This could deliver better insights in the long-term results of GLSS training and application.

## 6. Conclusions

Our research confirms that GLSS can be applied to make healthcare more sustainable in terms of environmental impact. Despite the study’s limited sample size from the Dutch healthcare setting, the inclusion of diverse participants enhances the generalizability of the findings to broader healthcare contexts. Projects addressed a variety of environmental areas including waste, travel, sustainable food and resource usage. Even though GLSS concepts and tools were considered valuable by the healthcare professionals participating in this study, seven barriers were identified that hindered GLSS implementation. These barriers could be taken into account by GLSS practitioners and healthcare organization management to improve future success of GLSS projects. It is important to further enhance the GLSS methodology based on practical experiences and theoretical developments to become even more suitable for making healthcare green. Future research should explore the long-term impact of GLSS training, compare its effectiveness with other improvement methods, and investigate organization-wide deployment to optimize its application in reducing the environmental impact of healthcare.

As healthcare has a significant impact on the environment, the results of this study suggest GLSS could be a suitable method to mitigate this impact and realize improvements by healthcare professionals themselves. Findings from this research can be used by GLSS trainers to improve education, and by healthcare professionals to enhance GLSS deployment programs and to optimize organizational settings for successful GLSS implementation.

## Figures and Tables

**Table 1:** Profile of individuals participating in case study.

	Number of Participants (Total *N* = 21)	Percentage of Participants
**Age**		
20–25	2	9.5%
26–35	9	42.9%
36–45	4	19.0%
45+	6	28.6%
**Gender at birth**		
Female	15	71.4
Male	6	28.6%
**Education level**		
Intermediate Vocational	1	4.8%
Higher Vocational	8	38.1%
Academic	12	57.1%
**Work organization**		
Academic hospital	15	71.4%
General hospital	3	14.3%
Nursing home	2	9.5%
Pharmaceutical company	1	4.8%
**Experience with process improvement**		
Low	12	57.1%
Medium	6	28.1%
High	3	14.3%

**Table 2:** Respondents’ demographics and background at start of training (t_0_) grouped by project status with variance between groups (ANOVA test result).

Question	All Participants (*N* = 21, 100%)	Subgroup			Variance between Groups (*p*-Value) ^1^
		Not Applied GLSS (*N* = 5, 24%)	Applied GLSS without Completing Project (*N* = 7, 33%)	Completed GLSS Project (*N* = 9, 43%)	
Age–Mean (SD)	37.1 (8.8)	38.2 (8.6)	35.3 (8.1)	37.9 (10.1)	0.815
Gender, N(%) male	6 (29%)	2 (40%)	0 (0%)	4 (44%)	-
Higher/Academic Education—N (% of group)	20 (95%)	5 (100%)	6 (86%)	9 (100%)	-
Involved in direct patient care—N (% of group)	10 (48%)	3 (60%)	5 (71%)	2 (22%)	-
Academic hospital—N (% of group)	14 (67%)	3 (60%)	4 (57%)	7 (78%)	-
Experience level process improvement ^2^	2.5 (1.0)	2.2 (0.5)	2.4 (1.0)	2.7 (1.3)	0.732

^1^ ANOVA test results for equal variances between groups (*p*-value); Null hypothesis: All means are equal; α = 0.05. ^2^ Answers are structured on a 5-point Likert scale from no experience (score 1) to very experienced (score 5).

**Table 3:** Respondents survey scores at end of training (t_1_) grouped by project status with variance between groups (ANOVA test result).

Question	All Participants (*N* = 21, 100%)	Subgroup			Variance between Groups (*p*-Value) ^1^
		Not Applied GLSS (*N* = 5, 24%)	Applied GLSS without Completing Project (*N* = 7, 33%)	Completed GLSS Project (*N* = 9, 43%)	
Enjoyed the training ^2^	4.1 (0.8)	4.5 (1.0)	4.2 (0.8)	3.9 (0.8)	0.468
Motivated to apply GLSS ^2^	4.3 (0.8)	3.7 (0.6)	4.2 (1.0)	4.7 (0.7)	0.173
Motivated to compete GLSS project ^2^	3.7 (1.3)	3.0 (1.8)	4.3 (1.0)	3.7 (1.2)	0.307
Training difficulty too high- N (% of group)	8 (38%)	2 (40%)	2 (29%)	4 (44%)	-

^1^ ANOVA test results for equal variances between groups (*p*-value); Null hypothesis: All means are equal; α = 0.05. ^2^ Answers are structured on a 5-point Likert scale from not enjoyed/motivated (score 1) to very much enjoyed/motivated (score 5).

**Table 4:** Respondents survey scores 6 to 8 months after training (t_2_) grouped by project status with variance between groups (ANOVA test result).

Question	All Participants (*N* = 21, 100%)	Subgroup			Variance between Groups (*p*-Value) ^1^
		Not Applied GLSS (*N* = 5, 24%)	Applied GLSS without Completing Project (*N* = 7, 33%)	Completed GLSS Project (*N* = 9, 43%)	
Q1. The training program inspired me to improve my achievement in making healthcare green ^2^	4.0 (0.8)	3.6 (0.5)	4.0 (0.9)	4.1 (0.9)	0.557
Q2. The training program increased my ability to perform well in my job role ^2^	3.2 (1.0)	3.2 (0.8)	2.7 (0.8)	3.6 (1.1)	0.244
Q3. While performing my job, my behavior changed after completing the program ^2^	3.0 (1.0)	3.2 (1.1)	2.7 (0.5)	3.2 (0.8)	0.564
Q4. I feel confident about applying what I have learned on the job ^2^	3.0 (0.9)	2.6 (1.1)	2.7 (0.5)	3.6 (0.9)	0.083
Number of GLSS tools applied	3.2 (3.7)	0 (0.0)	1.8 (0.9)	6 (4.2)	0.003
Number of GLSS tools expected to apply in future (SD)	7.6 (4.8)	3.8 (5.8)	7.4 (3.3)	9.8 (4.3)	0.076

^1^ ANOVA test results for equal variances between groups (*p*-value); Null hypothesis: All means are equal; α = 0.05. ^2^ Answers are structured on a 5-point Likert scale from strongly disagree (score 1) to strongly agree (score 5).

**Table 5:** GLSS tools evaluation by respondents based on current and anticipated use.

Participant ID	1	2	3	4	5	6	7	8	9	10	11	12	13	14	15	16	17	18	19	20	21
Project Charter	#	#	*	#	#	#	^	?	#	~	#	?	^	#	*	^	#	#	?	^	#
SIPOC	*	^	*	#	^	#	^	?	?	~	#	?	^	#	*	^	#	*	?	*	^
Critical to Quality Tree	*	^	*	#	*	*	^	?	?	~	?	?	*	#	*	*	?	~	?	^	^
Lifecycle Assessment	*	#	*	*	^	^	^	^	?	^	~	*	^	#	^	*	#	*	?	^	#
Process Mapping	*	#	*	*	*	^	^	?	?	~	^	^	^	^	^	*	?	*	?	^	*
DOTWIMP	*	*	*	*	^	^	^	?	*	~	~	?	~	#	^	*	?	*	?	^	#
5S	^	*	^	*	^	^	^	?	*	~	^	?	*	^	*	*	?	*	?	^	#
Value Add Analysis	^	#	*	~	?	^	^	?	*	~	^	?	*	~	*	*	#	~	?	^	^
Root Cause Analysis	^	#	#	^	^	^	^	?	?	~	#	?	^	*	^	*	#	^	?	#	^
Fishbone Diagram	*	^	#	^	*	^	^	?	?	~	#	*	^	*	^	*	?	^	?	^	#
Statistical analysis	^	#	*	~	?	^	^	?	~	?	?	*	*	#	^	~	?	*	?	#	*
Stakeholder analysis	^	^	*	*	*	^	^	?	~	*	#	^	*	#	#	^	#	#	?	#	#
Improvement plan	^	#	*	^	^	^	^	?	?	?	#	#	#	#	^	*	#	#	?	^	#
Control plan	^	^	*	^	^	^	^	?	?	?	?	^	#	*	^	*	?	#	?	^	#

Legend: # I applied this tool (dark green), ^ I strongly think I will apply this tool (light green), * I might apply this tool (yellow), ~ I don’t expect to apply this tool (orange), ? I don’t know (white/no color).

**Table 6:** Achievements in participant organization based on learnings from GLSS training (*N* = 16).

Achievement in Participant Organization	Frequency
Reduce use of products and/or materials (and waste)	7
Creating awareness on our impact on the environment	5
Green choices in procurement/ Replace disposable by reusable products	3
Cost reduction	3
Separation of waste types for recycling improved	3
Join Green Team	1
Energy reduction	1
Reduce travel	1
Reduction of waiting time	1
Reducing workload of employees	1
Reducing defects (errors/mistakes)	1

## Data Availability

The original contributions presented in the study are included in the article; further inquiries can be directed to the corresponding author/s.

## Public Involvement Statement

No public involvement in any aspect of this research.

## Guidelines and Standards Statement

This manuscript was drafted against the SRQR (Standards for reporting qualitative research; DOI: 10.1097/ACM.0000000000000388 (accessed on 9 March 2024)) for qualitative research.

## Appendix A. Training Content GLSS Training for Sustainable Healthcare

The training’s learning objectives are that after finishing the training, participants are able to:

- Describe the concepts, principles, and methodology of GLSS.
- Choose and apply tools and techniques from the GLSS toolset to a given case study to map, measure, analyze, improve, and control a process (Define, Measure, Analyze, Improve, and Control phases of a GLSS project).
- Choose an appropriate case from your work situation to apply GLSS to for the purpose of reducing climate impact.

**Table A1:** Tools included in the training.

DMAIC Phase	Green Lean Six Sigma Tool
Define	Stakeholder analysis
	Project charter
	Suppliers, input, process, outputs, and customers (SIPOC)
	Voice of the customer (VOC)
	Critical to quality (CTQ)
	Kano analysis
Measure	Lifecycle assessment (LCA)
	Process mapping
	Little’s Law
Analyze	Sort, straighten, shine, standardize, and sustain (5S)
	Forms of waste: defects, overproduction transportation waiting inventory, motion, and processing (DOTWIMP) and reduce, reuse, recycle (Ladder of Lansink)
	Value Add and Non-Value Add tool analysis
	Swimlane diagram
	Root cause analysis
	5 WHY
	Fishbone
	Brainstorming
	Affinity diagrams
Improvement	Improvement plan
	DPSIR framework: Driving forces, pressure, state, impacts and responses/improvements
	Reduce -> reuse -> remake -> recycle cycle
	Select solutions: single voting multi voting
	Implementation plan
Control	Control plan

## Appendix B. Description of Data Collected on t_0_ and t_1_

**Table A2:** Data collected at t0 (before starting the training).

Data Element	Data Input Format
Name	Free text
Age	Number
Gender at birth	M/F
Job function	Free text
Level of experience with process improvement	Likert scale 1 (no experience) to 5 (very experienced)
Healthcare institute	Free text
Highest education level completed	WO/HBO/MBO/Other: [free text]
How competent do you feel to implement “green” solutions at your workplace?	Likert scale 1 (not competent) to 5 (very competent)
Why do you want to start this training?	Free text
Entry test on GLSS concepts and tools	Multiple choice questions

Data Element

Data Input Format

**Table A3:** Data collected at t_1_ (directly after finishing training).

Data Element	Data Input Format
Did you enjoy taking the training?	Likert scale 1 (not at all) to 5 (very much)
How did you experience the difficulty of the training?	Likert scale 1 (very easy) to 5 (very difficult)
How motivated do you feel to apply GLSS at your workplace?	Likert scale 1 (not motivated at all) to 5 (very motivated)
Did you gain new knowledge from the training?	Likert scale 1 (not at all) to 5 (a lot of new knowledge)
How empowered do you feel to apply GLSS at your workplace?	Likert scale 1 (not empowered at all) to 5 (very empowered)
Knowledge test GLSS concepts and tools	Multiple choice questions
Written exam: Case study with assignment to describe approach to follow step by step	Open text

## Appendix C. Search Strategy Kirkpatrick Questionnaires

Search queries used in two databases searched are described below:

**Pubmed**
(Kirkpatrick[Title/Abstract]) AND (evaluation[Title/Abstract] OR assessment[Title/Abstract]) AND (questionnaire[Title/Abstract]) AND (behavior[Title/Abstract] OR behaviour[Title/Abstract] OR attitude[Title/Abstract])
**Education database (Proquest)**
ab(Kirkpatrick) AND ab(questionnaire) AND ab(evaluation OR assessment) AND ab(behavior OR behaviour OR attitude)

Inclusion criteria for article selection:

- Article written in English or Dutch.
- Population within the study should be students or members of the general, healthy population who take part in a training or other type of educational program.
- The article includes a questionnaire.
- The article questions in the questionnaire are applicable to Green Lean Healthcare training or can be made applicable by minor adjustment (e.g., change one or two words in the question).
- Article full text is accessible for researchers from the University of Amsterdam.

## Appendix D. Original and Modified Questions for Survey

The first column of the tables lists the original question from the specific source. If there are no sources for a question because it was conceived during review meetings, the purpose of the question is listed in the first column. The second column lists the modified version used in the present study. If the original question was used, it is indicated by *no changes* and if the question was not included, it is indicated by *not included*.

**Table A4:** Survey part A: evaluation of training according to Kirkpatrick’s theory. Answers are structured by use of a 5-point Likert scale from strongly disagree (score 1) to strongly agree (score 5).

Original Question	Adjusted Question Used in Final Questionnaire
Questions on Kirkpatrick’s Behavior level	
“The training programmes helped me to organise my role as head teacher more effectively”, Alsalamah and Callinan [16], p. 14.	Not included (not applicable for GLSS training)
“The training progammes inspired me to improve my achievement”, Alsalamah and Callinan [16], p. 14.	Q1. The GLSS training inspired me to improve my achievement in making healthcare green.
“The training progammes increased my ability to perform well in my job role”, Alsalamah and Callinan [16], p. 14.	Q2. The GLSS training increased my ability to perform well in my job role.
“The training progammes helped me to develop leadership behaviour”, Alsalamah and Callinan [16], p. 14.	Not included (not applicable for GLSS training)
“The training progammes developed some aspects of my behaviour”, Alsalamah and Callinan [16], p. 14.	Not included (removed after pretesting survey with one respondent due to length of survey and overlap with Q3)
“The training progammes helped me to prove myself in my work as head teacher”, Alsalamah and Callinan [16], p. 14.	Not included (not applicable for GLSS training)
“My job behaviour changed after completing the programme”, Alsalamah and Callinan [16], p. 14.	Q3. While performing my job, my behavior changed after completing the program.
No source.Purpose: Assess confidence of participant in application of learnings.	Q4. I feel confident about applying what I have learned on the job.
“What are the major concepts that you learned during this session?” Kirckpatrick & Kirckpatrick [15], p. 103.	Q5: What are the two major concepts that you learned during the GLSS training?
“What barriers do you anticipate that could limit your success at applying what you learned?” Kirckpatrick and Kirckpatrick [15], p. 103.	Q6: What barrier(s) did you experience that limited your success at applying GLSS in practice? - Selecting a suitable project/topic to work on - Lack of time - Lack of support from colleagues - Lack of support of management - Lack of confidence - I did not experience any barriers - GLSS is too complex - Other: …

**Table A5:** Survey part B: Lean Six Sigma Tools and project outcomes. Part B is divided into questions for participants who applied Lean Six Sigma tools in a real-life setting, e.g., during a project at their job and participants who did not.

Part B1.	
No source for question. Purpose: check if participant applied the learning tools in a real-life setting. If no they can skip the following questions and continue with X.	Q7. Did you apply learnings form the training in a real-life setting? Yes No
No source for question. Purpose: check category and which environmental impact problem the participant addressed in their projects.	Q8. Describe in a few sentences which root causes you addressed with solutions and the measurable impact (answer can be in Dutch) Open question
No source for question. Source WHO framework: Prats [18].	Q9. Which category fits the real-life setting where you applied the learnings best? A: Health workforce B: Water, sanitation and health care waste C: Energy D: Infrastructure, technologies and products
No source for question. Purpose: check which environmental impact problem participants addressed in their projects, check which outcome measures are applicable for describing results of their projects at an organization level.	Q10. What are the results you achieved within your organization by applying the learnings of the training? Creating awareness on our impact on the environment Reduce water use Reduce use of products/materials (and waste) Energy reduction Reducing of direct gas emissions (anesthetics or other) Reduce travel Reduce pollution of waste water Improve separation of waste types (gescheiden afvalverzameling) Green choices in procurement Lead Time reduction Cost reduction Reducing workload of employees Reducing errors/mistakes Reduction of waiting time None Other: ….
Part B2.	
No source for question. Purpose: check which GLSS tools are used/will be used.	Q11. For each of the following Lean Six Sigma tools, please indicate your expected usage (I applied this tool, I strongly think I will apply this tool, I might apply this tool, I don’t expect to apply this tool, I don’t know) Project charter SIPOC Critical to Quality Tree Lifecycle Assessment Process Mapping DOTWIMP 5S Value Add Analysis Root Cause Analysis Fishbone Diagram Statistical analysis Stakeholder analysis Improvement plan Control plan
No source for question. Purpose: check if other aspects of the training will be used/are used	Q12. Apart from the GLSS tools above, what are other things you learned and currently use in your job? (insights, concepts theory, best practice, reduce reuse recycle, new contacts) Open question
No source for question. Purpose: check if there are any items missing in the training	Q13. What are the tools that you missed in the training?

**Table A6:** Survey Part C: open questions. All based/inspired on the book Chapter 11 of Kirkpatrick and Kirkpatrick [15]. All questions are open questions.

“What do you see as the major reasons you took this course?” Kirkpatrick and Kirkpatrick ([14] p. 93) (Table 11.8).	No changes
“How can this program be improved? “ Kirkpatrick and Kirkpatrick ([14], p. 89) (Table 11.6).	Q14. What suggestions do you have to improve the training program?
“Please give an example of a positive outcome you have experienced since attending this training”, Kirkpatrick and Kirkpatrick ([14], p. 107).	Q15. Please mention any achievements you realized as a result of you following this training outside of you direct daily job (e.g., inspiring other departments, starting a green team.
“Overall, I am happy with how the program went? (Yes/No) Comments:” Kirkpatrick and Kirkpatrick ([14], p. 92) (Table 11.7).	No changes
“Please share any additional comments you may have?” Kirkpatrick and Kirkpatrick ([14], p. 89) (Table 11.6).	No changes

## References

[1] Van Daalen K.R., Romanello M., Rocklöv J., Semenza J.C., Tonne C., Markandya A., Dasandi N., Jankin S., Achebak H., Ballester J. (2022). The 2022 Europe Report of the Lancet Countdown on Health and Climate Change: Towards a Climate Resilient Future. Lancet Public Health.

[2] Trakulsunti Y., Antony J., Douglas J.A. (2020). Lean Six Sigma Implementation and Sustainability Roadmap for Reducing Medication Errors in Hospitals. TQM J..

[3] Zhu Q., Johnson S., Sarkis J. (2018). Lean Six Sigma and Environmental Sustainability: A Hospital Perspective. Supply Chain Forum Int. J..

[4] Farrukh A., Mathrani S., Taskin N. (2020). Investigating the Theoretical Constructs of a Green Lean Six Sigma Approach towards Environmental Sustainability: A Systematic Literature Review and Future Directions. Sustainability.

[5] McDermott O., Antony J., Bhat S., Jayaraman R., Rosa A., Marolla G., Parida R. (2022). Lean Six Sigma in Healthcare: A Systematic Literature Review on Motivations and Benefits. Processes.

[6] Rathi R., Vakharia A., Shadab M. (2022). Lean Six Sigma in the Healthcare Sector: A Systematic Literature Review. Mater. Today Proc..

[7] Garza-Reyes J.A. (2015). Green Lean and the Need for Six Sigma. Int. J. Lean Six Sigma.

[8] Cherrafi A., Elfezazi S., Govindan K., Garza-Reyes J.A., Benhida K., Mokhlis A. (2017). A Framework for the Integration of Green and Lean Six Sigma for Superior Sustainability Performance. Int. J. Prod. Res..

[9] Erdil N.O., Aktas C.B., Arani O.M. (2018). Embedding Sustainability in Lean Six Sigma Efforts. J. Clean. Prod..

[10] Gholami H., Jamil N., Mat Saman M.Z., Streimikiene D., Sharif S., Zakuan N. (2021). The Application of Green Lean Six Sigma. Bus. Strat. Env..

[11] Kaswan M.S., Rathi R., Garza-Reyes J.A., Antony J. (2023). Green Lean Six Sigma Sustainability—Oriented Project Selection and Implementation Framework for Manufacturing Industry. Int. J. Lean Six Sigma.

[12] Sijm-Eeken M., Jaspers M., Peute L., Mantas J., Gallos P., Zoulias E., Hasman A., Househ M.S., Charalampidou M., Magdalinou A. (2023). Training Healthcare Professionals to Mitigate the Climate Challenge—Development of a Lean Six Sigma E-Learning. Studies in Health Technology and Informatics.

[13] Maltby J., Williams G., Mcgarry J., Day L. (2014). Research Methods for Nursing and Healthcare.

[14] Kirkpatrick J.D., Kirkpatrick W.K. (2016). Kirkpatrick’s Four Levels of Training Evaluation.

[15] Alsalamah A., Callinan C. (2021). Adaptation of Kirkpatrick’s Four-Level Model of Training Criteria to Evaluate Training Programmes for Head Teachers. Educ. Sci..

[16] Kirkpatrick D.L., Brown S.M., Seidner C.J. (1998). The Four Levels of Evaluation. Evaluating Corporate Training: Models and Issues.

[17] Prats E.V. (2020). WHO Guidance for Climate-Resilient and Environmentally Sustainable Health Care Facilities.

[18] Cook J.V., Dickinson H.O., Eccles M.P. (2009). Response rates in postal surveys of healthcare professionals between 1996 and 2005: An observational study. BMC Health Serv Res..

[19] Yadav V., Kaswan M.S., Gahlot P., Duhan R.K., Garza-Reyes J.A., Rathi R., Chaudhary R., Yadav G. (2023). Green Lean Six Sigma for sustainability improvement: A systematic review and future research agenda. Int. J. Lean Six Sigma.

[20] Lee S.M., Lee D. (2022). Developing Green Healthcare Activities in the Total Quality Management Framework. Int. J. Environ. Res. Public Health.

[21] Letchumanan L.T., Gholami H., Yusof N.M., Ngadiman N.H.A.B., Salameh A.A., Štreimikienė D., Cavallaro F. (2022). Analyzing the Factors Enabling Green Lean Six Sigma Implementation in the Industry 4.0 Era. Sustainability.

[22] Mishra M.N. (2022). Identify Critical Success Factors to Implement Integrated Green and Lean Six Sigma. Int. J. Lean Six Sigma.

[23] Kumar S., Kumar N., Haleem A. (2015). Conceptualisation of Sustainable Green Lean Six Sigma: An Empirical Analysis. Int. J. Bus. Excell..

[24] Sijm-Eeken M., Jaspers M., Peute L. (2023). Identifying Environmental Impact Factors for Sustainable Healthcare: A Scoping Review. Int. J. Environ. Res. Public Health.

[25] Kotter J. (2012). Leading Change.

[26] Kubiak T.M. (2012). The Certified Six Sigma Master Black Belt Handbook.

[27] Jin H., Qu P., Zhou Y., Qu Q., Li H. (2024). Green Lean Six Sigma practice in the industrial and service sectors: From the systematic literature review to an integration diagram. Prod. Plan. Control..

[28] American Society for Quality (2014). The ASQ Certified CSSGB Six Sigma Green Belt Body of Knowledge.

[29] Kumari S., Gautam H., Nityadarshini N., Das B.K., Chaudhry R. (2021). Online Classes versus Traditional Classes? Comparison during COVID-19. J. Educ. Health Promot..

[30] Laux C., Mary E.J., Paul C. (2015). LSS Green Belt Projects Planned versus Actual Duration. J. Technol. Manag. Appl. Eng..

